# Proteomic profiling reveals that collismycin A is an iron chelator

**DOI:** 10.1038/srep38385

**Published:** 2016-12-06

**Authors:** Makoto Kawatani, Makoto Muroi, Akira Wada, Gyo Inoue, Yushi Futamura, Harumi Aono, Kenshirou Shimizu, Takeshi Shimizu, Yasuhiro Igarashi, Naoko Takahashi-Ando, Hiroyuki Osada

**Affiliations:** 1 Chemical Biology Research Group, RIKEN CSRS, 2-1 Hirosawa, Wako, Saitama 351-0198, Japan; 2 Nonnatural Amino Acid Technology Team, RIKEN CLST, 1-7-22 Suehiro-cho, Tsurumi-ku, Yokohama, Kanagawa 230-0045, Japan; 3 Department of Applied Chemistry, Faculty of Science and Engineering, Toyo University, 2100 Kujirai, Kawagoe, Saitama 350-0815, Japan; 4 Department of Materials Science and Engineering, Graduate School of Engineering, Tokyo Denki University, 5 Senju Asahi-cho, Adachi-ku, Tokyo 120-8551, Japan; 5 Biotechnology Research Center and Department of Biotechnology, Toyama Prefectural University, 5180 Kurokawa, Imizu, Toyama 939-0398, Japan

## Abstract

Collismycin A (CMA), a microbial product, has anti-proliferative activity against cancer cells, but the mechanism of its action remains unknown. Here, we report the identification of the molecular target of CMA by ChemProteoBase, a proteome-based approach for drug target identification. ChemProteoBase profiling showed that CMA is closely clustered with di-2-pyridylketone 4,4-dimethyl-3-thiosemicarbazone, an iron chelator. CMA bound to both Fe(II) and Fe(III) ions and formed a 2:1 chelator-iron complex with a redox-inactive center. CMA-induced cell growth inhibition was completely canceled by Fe(II) and Fe(III) ions, but not by other metal ions such as Zn(II) or Cu(II). Proteomic and transcriptomic analyses showed that CMA affects the glycolytic pathway due to the accumulation of HIF-1α. These results suggest that CMA acts as a specific iron chelator, leading to the inhibition of cancer cell growth.

Bioactive natural products are important sources of pharmaceutical leads in medicine and bioprobes in chemical biology for the exploration of biological functions^1^,^2^. They are often found by cell-based screens; however, identification of the cellular targets of bioactive natural products is a time-consuming step in the drug development process. There are two fundamentally different approaches to identify molecular targets of the bioactive small molecules: affinity-based direct approaches and phenotype-based indirect approaches^3^,^4^. Affinity purification with small-molecule probes is the most common approach, but such direct approaches are based on the assumption that the target of the small molecule is a protein^4^,^5^. Phenotype-based approaches, on the other hand, compare the biological profiles of small molecules of interest and known reference drugs, *e.g.*, the NCI60 cell screen^6^, the JFCR39 cell line panel^7^, and cell morphological profiling^8^. We have developed a proteome-based profiling approach, named ChemProteoBase, to predict small-molecule targets using two-dimensional fluorescence differential gel electrophoresis (2D-DIGE)^9^. This system is based on recording the comprehensive patterns of variation in proteins in HeLa cells that are treated with small molecules. Using this system, we have successfully identified the targets of various bioactive small molecules, such as natural product pyrrolizilactone and natural product derivative BNS-22^3^,^10^,^11^,^12^.

Collismycin A (CMA), a natural product produced by *Streptomyces* sp., is an antibiotic and has cytotoxic activity against cancer cells^13^,^14^,^15^,^16^. In this study, we use ChemProteoBase profiling to show that collismycin A acts as an iron chelator^17^. Iron is an essential element for all organisms, and iron-requiring proteins play a crucial role in a variety of cellular processes, such as energy metabolism, DNA synthesis, DNA repair, cell cycle progression, epigenetic regulation, and response to hypoxia^18^,^19^,^20^. At the biological level, iron exists in two oxidation states: ferrous iron, Fe(II) and ferric iron, Fe(III). The ability to go from one state to the other through the acceptance or donation of an electron is a key factor that helps in a variety of biological functions. In addition, free iron can generate reactive oxygen species (ROS) through the Fenton reaction, resulting in DNA, protein, and lipid damage^19^.

Recent studies have shown that iron can contribute to tumor initiation, progression, and metastasis, and iron regulatory pathways are perturbed in many tumors^20^,^21^. Consequently, the iron chelation strategy has shown promise in providing new options in cancer chemotherapy. Deferoxamine (DFO), deferasirox, and deferiprone, which are commercially-approved drugs that were initially developed for the treatment of iron overload, have shown anti-proliferative activity against a wide variety of tumors^22^. In addition, many other iron chelators have been developed that are at various stages of clinical and preclinical testing. These include triapine, pyridoxal isonicotinoyl hydrazone (PIH), and di-2-pyridylketone thiosemicarbazones (DpT), such as di-2-pyridylketone 4,4-dimethyl-3-thiosemicarbazone (Dp44mT)^23^,^24^,^25^.

Our data indicate that CMA acts as a specific iron chelator in cells, as predicted by ChemProteoBase profiling. CMA binds to both Fe(II) and Fe(III) ions and forms 2:1 chelator-iron complex that inactivates the iron ion, resulting in the inhibition of cancer cell growth.

## Results

### CMA inhibits cancer cell growth and causes G1 cell cycle arrest

**Figure 1:**
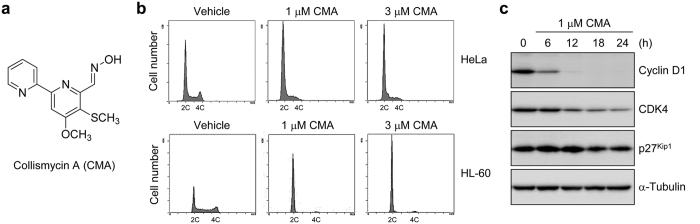
CMA arrests cell cycle at the G1 phase. (**a**) Structure of CMA. (**b**) Effect of CMA on the cell cycle. HeLa or HL-60 cells were treated with the indicated concentrations of CMA for 12 h, and then analyzed by flow cytometry after propidium iodide staining. (**c**) CMA decreases the expression of cyclin D1. HeLa cells were treated with 1 μM CMA for the indicated times. Cell lysates were immunoblotted with anti-cyclin D1, anti-CDK4, anti-p27^Kip1^, and anti-α-tubulin.

We first examined the growth inhibitory effects of CMA (Fig. 1a) against human cancer cell lines, and found that CMA inhibits their growth with IC_50_ values ranging from 0.1 to 0.4 μM for 72 h (Table 1). When HeLa cells or HL-60 cells were treated with CMA for 12 h, G1 phase population increased significantly (Fig. 1b). This effect was reversible as the cell cycle arrest was canceled by the depletion of CMA from culture media (Supplementary Fig. S1). Western blot analysis after incubation with CMA demonstrated that the expression of cyclin D1 was markedly decreased in a time-dependent manner (Fig. 1c). The expression rates of cyclin D1 after treatment of CMA decreased to 44.4% (6 h, *P* < 0.001), 12.2% (12 h, *P* < 0.001), 7.7% (18 h, *P* < 0.001), and 7.1% (24 h, *P* < 0.001) (Supplementary Fig. S2a). CDK4 was also decreased after 12 h treatment of CMA (Fig. 1c). The expression rates of CDK4 after treatment of CMA decreased to 67.3% (12 h, *P* < 0.001), 38.3% (18 h, *P* < 0.001), and 30.1% (24 h, *P* < 0.001) (Supplementary Fig. S2b). On the other hand, the expression of p27^Kip1^ was not changed significantly (Fig. 1c and Supplementary Fig. S2c). Thus, CMA leads to cell cycle arrest at the G1 phase with a marked decrease of cyclin D1.

### Target prediction of CMA by ChemProteoBase profiling

**Figure 2:**
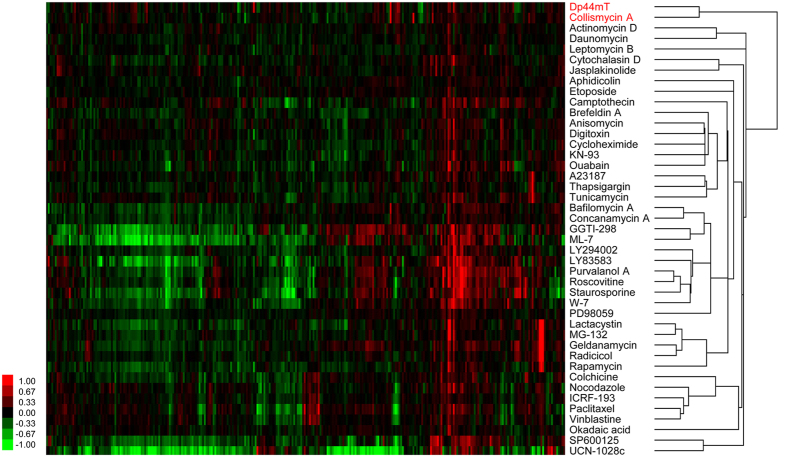
Cluster analysis by ChemProteoBase profiling. HeLa cells were treated with 5 μM CMA for 18 h, and proteomic analysis was performed by the 2-D DIGE system. Quantitative data of the 296 common spots (x-axis) derived from CMA and those of 42 well-characterized compounds were analyzed by hierarchical clustering. In the heat map, log-fold (natural base) of the normalized volume is shown on the colored scale.

To identify the molecular target of CMA, we performed ChemProteoBase profiling. HeLa cells were treated with 5 μM CMA for 18 h, and the resulting cell lysates were subjected to 2D-DIGE. The proteomic variation of 296 spots that matched on all gel images was quantified, followed by hierarchical cluster analysis and calculation of cosine similarities of compounds in the database against CMA. Hierarchical cluster analysis with 42 standard compounds showed that CMA clustered with Dp44mT^26^,^27^, a synthetic iron chelator (Fig. 2). Similarity analysis showed that Dp44mT ranked in the highest position among 143 compounds contained in the ChemProteoBase (cosine similarity = 0.71, Table 2). These data suggest that CMA is an iron chelator.

### CMA forms 2:1 chelator-iron complex

**Figure 3:**
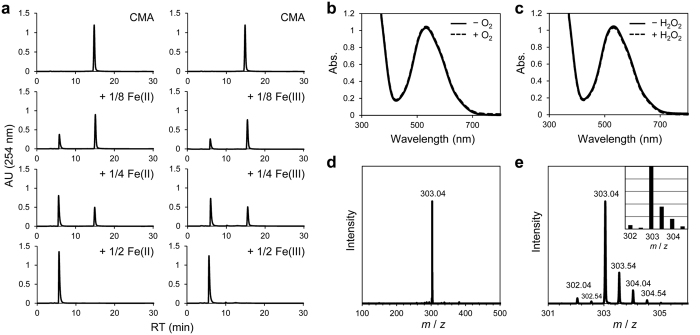
CMA forms 2:1 chelator-iron complex. (**a**) CMA binds to both Fe(II) and Fe(III) ions. The mixed solutions of CMA with various concentrations of Fe(II) or Fe(III) ion were analyzed by HPLC. (**b**) Absorption spectra in the formation of Fe(II)-(CMA)_2_ complex (solid line) and after the reaction of the complex with O_2_ (dashed line). (**c**) Absorption spectra in the formation of Fe(II)-(CMA)_2_ complex (solid line) and after the reaction of the complex with H_2_O_2_ (dashed line). (**d**,**e**) ESI-mass spectra of isolated Fe(II)-(CMA)_2_ complex (inset: calculated isotope pattern of positive ion clusters at *m/z* 303.0).

To confirm whether CMA forms an iron complex, we performed qualitative analysis using High Performance Liquid Chromatography (HPLC). When CMA was mixed with Fe(II) or Fe(III) ions at half the concentration of CMA, the peak of CMA shifted completely (Fig. 3a). Furthermore, we carried out absorption spectroscopic analysis on the reactions of CMA with Fe(II) and Fe(III) ions, respectively. The binding of CMA to Fe(II) ion induced a new intense absorption with a maximum at 535 nm. The intensity increased linearly upon incremental concentration of Fe(II) ion and saturated at 0.5 equiv. with respect to CMA (Supplementary Fig. S3). This result clearly demonstrates a rapid formation of Fe(II)-(CMA)_2_ complex, which is identical to that obtained by HPLC analysis (Fig. 3a: left). Moreover, upon mixing of CMA and Fe(III) ion of 0.5 equiv. with respect to the chelator, a new broad absorption in the range from 360 nm to 420 nm increased immediately (Supplementary Fig. S4). This spectral pattern, which is similar to that observed in the formation of Fe(III)-(Dp44mT)_2_ complex^28^, and the similarity between Fig. 3a (left) and Fig. 3a (right) in HPLC analysis suggest that CMA is also able to bind to Fe(III) ion and form Fe(III)-(CMA)_2_ complex.

We next analyzed the reactivity of Fe(II)-(CMA)_2_ complex to O_2_ and H_2_O_2_, respectively, by using absorption and ESI-mass spectroscopic measurements. The intense absorption at 535 nm characteristic of the ferrous complex (Fig. 3b: solid line) was not changed at all even after exposing the solution to the atmosphere (Fig. 3b: dashed line). This phenomenon indicates that Fe(II)-(CMA)_2_ complex did not react with O_2_ at ambient temperature. Moreover, a purple powder was isolated from the solution. ESI-mass spectroscopic measurement of the powder redissolved in solution showed positive ion peak clusters at *m/z* 303.04, whose observed mass and isotope pattern corresponded to the [Fe(CMA)_2_]^2+^ ion (Fig. 3d,e). This observation reveals the presence of a stable Fe(II) complex, formulated as [Fe(CMA)_2_]^2+^, in solution. In addition, as shown in Fig. 3c, the absorption spectrum derived from [Fe(CMA)_2_]^2+^ complex was unchanged in the presence of an excess amount of H_2_O_2_ (20 eq./Fe(II) ion), demonstrating that [Fe(CMA)_2_]^2+^ complex did not exhibit the high reactivity with H_2_O_2_ required to facilitate the Fenton reaction.

### CMA acts as a specific iron chelator in cells

**Figure 4:**
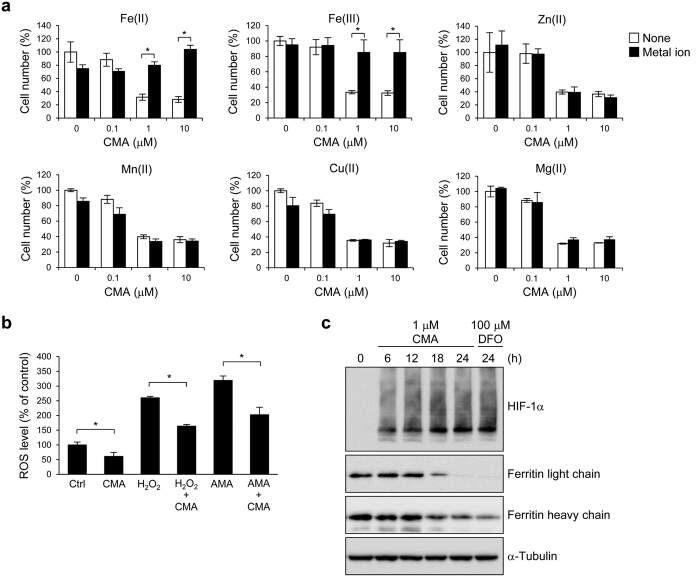
CMA acts as a specific iron chelator. (**a**) The addition of Fe(II) and Fe(III) ions rescue cells from CMA-induced growth inhibition. HeLa cells were treated with the indicated concentrations of CMA in the presence or absence of 10 μM FeCl_2_, 10 μM FeCl_3_, 10 μM ZnCl_2_, 1 μM MnCl_2_, 1 μM CuCl_2_, or 10 μM MgCl_2_ for 72 h, and cell growth was analyzed by WST-8 assay. Data are shown as the mean ± SD (n = 3). Statistical analysis was performed by using ANOVA followed by Tukey-Kramer test. *P < 0.01. (**b**) CMA decreases intracellular ROS level. HL-60 cells were pretreated with 1 μM CMA for 30 min and were treated with 1 mM H_2_O_2_ or 100 nM antimycin A (AMA) for 1 h. The ROS level was measured by flow cytometry using carboxy-H2DCF-DA labeling. Data are shown as the mean ± SD (n = 3). Statistical analysis was performed by using ANOVA followed by Tukey-Kramer test. *P < 0.01. (**c**) CMA markedly induces the expression of HIF-1α. HeLa cells were treated with 1 μM CMA or 100 μM DFO for the indicated times. Cell lysates were immunoblotted with anti-HIF-1α, anti-ferritin light chain, anti-ferritin heavy chain, and anti-α-tubulin.

If CMA acts as an iron chelator and causes the inhibition of cell growth, the addition of exogenous iron ion should abolish the biological activity of CMA. As shown in Fig. 4a, CMA-induced cell growth inhibition was completely canceled by Fe(II) and Fe(III) ions, but not by other metal ions, including Zn(II), Mn(II), Cu(II), and Mg(II). To confirm whether CMA binds to Cu or Zn ions to inhibit cancer cell growth, we assessed the cytotoxic activity of CMA in the presence of TM (tetrathiomolybdate) as a Cu chelator^29^ or TPEN [N,N,N′,N′-tetrakis(2-pyridylmethyl)ethylenediamine] as a Zn chelator^30^. As shown in Supplementary Fig. S5, the activity of CMA was not reduced at all even if the concentrations of TM and TPEN were higher than that of CMA.

Iron is involved in many cellular processes, including DNA synthesis, cell cycle regulation, energy generation, ROS generation, and hypoxia-inducible factor-1 (HIF-1) and WNT pathways^20^. When HL-60 cells were treated with 1 μM CMA for 1.5 h, endogenous ROS level was significantly decreased (Fig. 4b). In addition, pretreatment with 1 μM CMA also inhibited increased ROS level induced by H_2_O_2_ or antimycin A, an inhibitor of mitochondrial electron transport (Fig. 4b). Furthermore, CMA as well as DFO, a known iron chelator, markedly increased the expression of HIF-1α protein (Fig. 4c and Supplementary Fig. S6). The expression levels of HIF-1α after treatment of CMA increased 10.9-fold (6 h, *P* < 0.01), 15.8-fold (12 h, *P* < 0.001), 15.7-fold (18 h, *P* < 0.001), and 12.3-fold (24 h, *P* < 0.001) (Supplementary Fig. S7a). The expression level of HIF-1α after treatment of DFO increased 13.4-fold (24 h, *P* < 0.001) (Supplementary Fig. S7a). CMA as well as DFO significantly decreased the expression of ferritin, which is an intracellular iron storage protein (Fig. 4c). The expression rates of ferritin light chain after treatment of CMA decreased to 69.3% (12 h, *P* < 0.05), 32.7% (18 h, *P* < 0.001), and 12.6% (24 h, *P* < 0.001) (Supplementary Fig. S7b). The expression rate of ferritin light chain after treatment of DFO decreased to 11.0% (24 h, *P* < 0.001) (Supplementary Fig. S7b). The expression rates of ferritin heavy chain after treatment of CMA decreased to 74.3% (6 h, *P* < 0.001), 75.6% (12 h, *P* < 0.001), 54.5% (18 h, *P* < 0.001), and 46.9% (24 h, *P* < 0.001) (Supplementary Fig. S7c). The expression rate of ferritin heavy chain after treatment of DFO decreased to 39.0% (24 h, *P* < 0.001) (Supplementary Fig. S7c). These results suggest that CMA acts as a specific iron chelator, resulting in the inhibition of cancer cell growth.

### Proteomic and transcriptomic analyses of CMA-treated cells

Our ChemProteoBase predicts drug targets by comparing just the spot patterns without requiring any protein information. However, we have identified about 90% of the protein spots used in the profiling so far. Such protein information enables the investigation of the mode of action of the compound on a proteomic level. Keyword based analysis of CMA-induced proteomic changes showed that metabolic pathway—especially glycolysis and oxidative phosphorylation—related proteins and oxidative stress-related proteins underwent changes (Supplementary Fig. S8). In particular, most of glycolysis-related proteins were upregulated and oxidative phosphorylation-related proteins were downregulated (Supplementary Fig. S8). As shown in Table 3, glycolysis-related proteins, such as fructose-bisphophate aldolase A (ALDOA), phosphoglycerate kinase 1 (PGK1), phosphoglycerate mutase 1 (PGAM1), and triosephosphate isomerase (TPI1), were significantly upregulated in HeLa cells treated with CMA as well as Dp44mT. We further performed transcriptome analysis and found that all these proteins were upregulated at the mRNA level (Table 3), suggesting that these proteomic changes are mainly regulated by gene expression but not by protein modification. Also, iron-related proteins, such as procollagen-lysine, 2-oxoglutarate 5-dioxygenase 2 (PLOD2) and prolyl 4-hydroxylase subunit α-1 (P4HA1), were markedly upregulated in HeLa cells treated with CMA as well as Dp44mT (Table 3).

## Discussion

In this study, we showed that CMA acts as an iron chelator, resulting in the inhibition of cancer cell growth. CMA binds to both Fe(II) and Fe(III) ions and forms 2:1 chelator-iron complex (Fig. 3, Supplementary Fig. S3, and Supplementary Fig. S4). Considering the structure of CMA (Fig. 1a) and the property of the iron ion, the complex may have an octahedral configuration with the iron ion in the middle, in which three nitrogen atoms from pyridine and oxime groups of CMA could be ligands. CMA is able to capture an Fe(II) ion instantly to form a stable Fe(II) complex with extremely low reactivity to O_2_ and H_2_O_2_ (Fig. 3b). Therefore, the observed inhibition of the growth of various cancer cells (Table 1) could be due to CMA lowering the total concentration of iron ions below what is necessary for cell proliferation and cell cycle and preventing the generation of ROS through reaction with O_2_ or H_2_O_2_. In fact, CMA decreased intracellular ROS level (Fig. 4b), suggesting that CMA inhibits the Fenton reaction, in which Fe(II) ion reacts with hydrogen peroxide to produce the hydroxyl radical, the most reactive ROS. Thus, CMA is a chelator that inactivates the iron ions.

The inhibitory effect of CMA on cell growth was completely canceled by iron ions but not by other metal ions (Fig. 4a), suggesting that CMA acts as a specific iron chelator in cells, and the CMA-iron complex itself has no biological effect, at least against cell proliferation. Because iron is involved in many cellular processes, such as DNA synthesis, energy metabolism, and cell cycle progression, CMA must affect various iron-related proteins and processes directly or indirectly. Because CMA arrested cell cycle at the G1 phase, the main target of CMA may be ribonucleotide reductase, which is a key enzyme in DNA synthesis for the conversion of ribonucleotides into deoxyribonucleotides, and has two iron atoms in its active site^31^.

Our ChemProteoBase can not only predict the molecular targets of bioactive small molecules by comparing protein variation patterns but can also explore their biological effects by using information from each of the studied proteins. Keyword based analysis of CMA-induced proteomic changes showed that glycolysis-related proteins tend to be upregulated and oxidative phosphorylation-related proteins tend to be downregulated (Supplementary Fig. S8). This could be due to the accumulation of HIF-1α consequent to CMA treatment (Fig. 4c and Supplementary Fig. S7a). HIF-1α is known to upregulate glycolysis and downregulate oxidative phosphorylation to help cells survive in a low oxygen (hypoxic) environment^32^,^33^. In normoxic conditions, HIF-1α undergoes hydroxylation by HIF prolyl hydroxylases. The hydroxylation allows the binding of von Hippel-Lindau protein (pVHL) to HIF-1α and subsequently results in the ubiquitination of the protein that leads to proteasomal degradation^34^,^35^. Because HIF prolyl hydroxylases require iron as a cofactor, CMA may inhibit the activity of HIF prolyl hydroxylases, leading to the accumulation of HIF-1α. ALDOA, PGK1, PGAM1, and TPI1, as well as P4HA1, which were significantly upregulated by CMA, have been reported to be HIF-1 target genes^36^,^37^.

A number of iron chelators have been designed and studied as anticancer agents in various types of cancer^25^,^38^. Importantly, every chemical property of a chelator, including binding potency and selectivity for metal ion, redox potential, charge, solubility, lipophilicity, pH dependence, and kinetics, can influence the biological outcome^39^. For example, Dp44mT can bind to copper as well as iron^29^. Treatment with Dp44mT causes efflux of iron from cells, but has the opposite effect on copper, which accumulates within lysosomes due to their acidic pH and the ionization characteristics of Dp44mT. Dp44mT forms a redox-active copper complex that promotes ROS generation, which degrades the lysosomal membrane and subsequently leads to apoptosis^29^. Although further investigations are needed, especially to understand in more detail the mechanism of action and *in vivo* antitumor efficacy, CMA can be used to understand iron metabolic pathways and can be administered as an anticancer agent in cancer chemotherapy. In addition, because iron is also involved in the pathophysiology of a number of diseases including malaria, HIV AIDS, fungal infections, and neurodegenerative diseases^38^, development of new iron chelators would be advantageous in a number of clinical scenarios. In conclusion, we have demonstrated that CMA acts as an specific iron chelator that binds to both Fe(II) and Fe(III) ions, resulting in the inhibition of cancer cell growth. Our study also shows that ChemProteoBase is useful for target prediction of bioactive small molecules, especially nonprotein-targeting small molecules.

## Methods

### Materials

CMA was isolated from the culture broth of *Streptomyces* sp. as described elsewhere^13^,^14^. Dp44mT, deferoxamine mesylate salt, ammonium tetrathiomolybdate (TM), and N,N,N′,N′-tetrakis(2-pyridylmethyl) ethylenediamine (TPEN) were purchased from Sigma-Aldrich. Antimycin A was obtained from the RIKEN Natural Products Depository (NPDepo)^40^.

### Cell culture

The human cancer cell lines HeLa, A549, HT-1080, and A431 cells were cultured in Dulbecco’s modified Eagle’s medium (DMEM) (Invitrogen) containing 10% fetal calf serum (Sigma-Aldrich), 50 units/mL penicillin G (Gibco) and 50 μg/mL streptomycin (Gibco). HL-60, K562, Jurkat, MKN74, MCF-7, and HT-29 cells were cultured in RPMI 1640 (Invitrogen) containing 10% fetal calf serum, 50 units/mL penicillin G and 50 μg/mL streptomycin. All cell lines were incubated at 37 °C in a humidified atmosphere containing 5% CO_2_.

### Cell proliferation assay

Cell proliferation was determined by using a Cell Count Reagent SF (Nacalai Tesque, Kyoto, Japan) according to the manufacturer’s instructions. Briefly, cells were seeded in a 96-well culture plate, cultured overnight, and exposed to compound for 72 h. Then, 1/20 volume of WST-8 solution was added to each well, and the plates were incubated at 37 °C for 1 h. Cell proliferation was measured as the absorbance at 450 nm on a microplate reader (Perkin Elmer).

### Cell cycle analysis

Cells were treated with compound for the indicated times. Cells were then harvested, washed with PBS and fixed in 70% ethanol. Cells were washed twice with PBS and incubated with 50 μg/mL propidium iodide in PBS containing 2 μg/mL RNase A (Nacalai Tesque) for 30 min. The DNA content of the cells was analyzed on a Cytomics FC500 flow cytometer (Beckman Coulter).

### Western blot

Western blot was performed as described with slight modifications^41^. Briefly, cells were lysed by sonication in RIPA buffer (25 mM HEPES, pH 7.8, 0.5 M NaCl, 5 mM EDTA, 1.5% Triton X-100, 1.0% sodium deoxycholate and 0.1% SDS), supplemented with a protease inhibitor cocktail (Roche). After protein concentration measurement, samples were subjected to SDS-PAGE, transferred onto a PVDF membrane (Millipore), and immunoblotted with anti-cyclin D1 (#2922, Cell Signaling Technology), anti-CDK4 (sc-601, Santa Cruz Biotechnology), anti-p27^Kip1^ (610242, BD Biosciences), anti-α-tubulin (T9026, Sigma-Aldrich), anti-HIF-1α (610958, BD Biosciences), anti-ferritin light chain (sc-74513, Santa Cruz Biotechnology), or anti-ferritin heavy chain (sc-376594, Santa Cruz Biotechnology). Band intensities were quantified using Fusion Solo S System (Vilber-Lourmat).

### ChemProteoBase profiling

ChemProteoBase profiling was performed as described previously^9^. Briefly, HeLa cells were treated with 5 μM CMA for 18 h. Proteome analysis of the cell lysate was performed by using the 2D difference gel electrophoresis (2D-DIGE) system (GE Healthcare), and images of the gels were analyzed with Progenesis SameSpots (Nonlinear Dynamics). Of more than 1000 spots detectable in each 2D gel, 296 variational spots were found to be common between gels of reference-compound-treated cells and were selected as described previously^9^. Next, the volume of each spot was normalized by using the average of the corresponding control values from DMSO-treated control cells. From the normalized volumes of the 296 spots, cosine similarity between compounds was calculated, and hierarchical clustering analysis was performed using Cluster 3.0 (clustering method; centroid linkage with the means of uncentred correlation). The predictive dendrogram was visualized using Java Treeview 1.1.3.

### Analysis of CMA-iron complex

For HPLC analysis, CMA dissolved in MeOH was mixed with various concentrations of FeCl_2_ or FeCl_3_ solution, and analyzed by HPLC (Waters 996). The HPLC conditions were as follows: column, SSC Senshu Pak Pegasil ODS 4.6 ϕ × 250 mm; pattern, isocratic; flow rate, 1 mL/min; solvent, 35% MeCN – 5 mM CH_3_COONH_4_. UV detection was performed at 254 nm.

For absorption and ESI-mass spectroscopic measurements, Fe(II)-(CMA)_2_ complex was generated by mixing CMA and FeSO_4_ at a molar ratio of 2:1 in MOPS buffer (20 mM MOPS, 100 mM NaCl, pH 7.4, 5% DMSO) degassed of dissolved O_2_. The reactions of the corresponding Fe(II)-(CMA)_2_ complex with O_2_ and H_2_O_2_ were carried out by introduction of O_2_ into the solution of the ferrous complex with gentle bubbling and addition of H_2_O_2_ (20 eq./Fe(II) ion) to the solution, respectively. The spectral changes were analyzed by using V630 spectrometer (Jasco). The purple powder of Fe(II)-(CMA)_2_ complex was obtained from the solution under Ar atmosphere or anaerobic conditions after the absorption analysis. The formulation and purity of the isolated complex were identified by ESI mass spectrophotometer (QSTAR Elite, AB SCIEX). The sample for the ESI mass spectroscopic measurement was prepared in MeOH. MS (ESI, *m/z*) 303.04 [Fe(CMA)_2_]^2+^.

### ROS assay

HL-60 cells were seeded in 24-well plates, cultured overnight, and treated with compounds. Then, cells were incubated in PBS containing 10 mM carboxy-2′,7′-dichlorodihydrofluorescein diacetate (carboxy-H_2_DCF-DA) (Life Technologies) for 30 min at 37 °C, washed with PBS and immediately analyzed using a Cytomics FC500 flow cytometer (Beckman Coulter).

### Transcriptome analysis

HeLa cells were seeded in 10-cm culture dishes, cultured overnight, and treated with 5 μM CMA or 5 μM Dp44mT for 18 h. Then, cells were harvested and total RNA from the cells was prepared using Isogen (Nippon Gene, Japan) according to the manufacturer’s instructions. Gene chip analysis was performed using a GeneChip Human Genome U133 Plus 2.0 Array (Affymetrix). The DNA microarray data of CMA- or Dp44mT-treated HeLa cells have been deposited in NCBI’s Gene Expression Omnibus and are accessible through GEO Series accession number GSE78052.

## Additional Information

**How to cite this article**: Kawatani, M. *et al*. Proteomic profiling reveals that collismycin A is an iron chelator. *Sci. Rep.* **6**, 38385; doi: 10.1038/srep38385 (2016).

**Publisher's note:** Springer Nature remains neutral with regard to jurisdictional claims in published maps and institutional affiliations.

## Supplementary Material

Supplementary Information

## Figures and Tables

**Table 1:** Effect of CMA on cell growth in various cancer cell lines.

Cell line	Origin	IC_50_ (μM)
HeLa	Cervical cancer	0.30 ± 0.18
HL-60	Leukemia	0.29 ± 0.13
K562	Leukemia	0.34 ± 0.14
Jurkat	Lymphoma	0.13 ± 0.02
A549	Non-small-cell lung cancer	0.16 ± 0.04
MKN74	Gastric cancer	0.22 ± 0.06
MCF-7	Breast cancer	0.25 ± 0.03
HT-29	Colon cancer	0.42 ± 0.01
HT-1080	Fibrosarcoma	0.067 ± 0.016
A431	Squamous cancer	0.19 ± 0.02

Cells were treated with CMA for 72 h, and cell growth was analyzed by WST-8 assay. Data are shown as the mean ± SD (n = 3).

**Table 2:** Similarity ranking for CMA by ChemProteoBase profiling.

Rank	Similarity	Compound
1	0.71	Dp44mT
2	0.42	Mizoribine
3	0.37	A23187
4	0.37	5-FU
5	0.36	Aphidicolin
6	0.35	Concanamycin A
7	0.34	Trichostatin A
8	0.33	Etoposide
9	0.33	Bafilomycin A
10	0.32	Methotrexate

Cosine similarity between CMA and each compound in ChemProteoBase (143 compounds) was calculated. The top ten compounds similar to CMA in ranking are displayed.

**Table 3:** Proteomic and transcriptomic changes of the spots that changed significantly in the proteomic analysis of CMA-treated HeLa cells.

Protein name	GENE		Proteomic changes					Transcriptomic changes			Remarks
			Spot no	CMA		Dp44mT			CMA	Dp44mT	
Adenylyl cyclase-associated protein 1	CAP1		1137	0.54	^***^	0.76	^**^		1.05	1.34	
Annexin A1	ANXA1		1491	1.14	^**^	1.12	^**^		2.88	2.77	
ATP-dependent RNA helicase DDX3X	DDX3X		2064	1.23	^**^	1.20	^**^		0.97	1.06	
Cytoplasmic aconitate hydratase	ACO1		662	1.32	^***^	1.09			1.21	0.99	Iron
Dihydropyrimidinase-related protein 3	DPYSL3		890	1.48	^**^	1.12			0.91	0.92	
DNA polymerase subunit delta 2	POLD2		1237	1.25	^**^	1.08			0.74	0.70	
Elongation factor 1-beta	EEF1B2		2039	0.85	^*^	0.98			0.90	0.94	
Elongation factor 1-delta	EEF1D		1556	1.20	^***^	1.19	^***^		0.99	0.99	
Elongation factor 2	EEF2		643	1.87	^***^	1.12			1.05	1.00	
			657	0.75	^**^	0.53	^***^				
Fructose-bisphosphate aldolase A	ALDOA		2091	1.44	^***^	1.28	^**^		1.78	1.52	Glycolysis
			2132	1.27	^***^	1.34	^***^				
Guanine nucleotide-binding protein subunit beta 2-like 1	GNB2L1		1627	0.76	^***^	0.76	^***^		0.71	0.70	
Heat shock cognate 71 kDa protein	HSPA8		1980	0.73	^***^	0.91	.		0.49	0.73	
Heat-shock protein beta-1	HSPB1		1764	0.72	^***^	0.84	^***^		0.74	0.79	
			2212	0.72	^***^	0.77	^***^				
Inosine-5′-monophosphate dehydrogenase 2	IMPDH2		2150	0.76	^**^	0.87	^*^		0.78	0.49	
Nucleophosmin	NPM1		2026	0.51	^***^	0.84	^*^		0.84	0.76	
Peroxiredoxin-2	PRDX2		1857	1.23	^***^	1.20	^***^		1.27	1.20	
Peroxiredoxin-6	PRDX6		1762	0.69	^***^	0.90	^**^		0.61	0.57	
Phosphoglycerate kinase 1	PGK1		1373	1.63	^***^	1.41	^***^		2.78	2.37	Glycolysis
Phosphoglycerate mutase 1	PGAM1		1745	1.39	^***^	1.29	^**^		1.39	1.57	Glycolysis
Prelamin-A/C	LMNA		840	1.37	^***^	1.46	^***^		1.30	1.22	
			845	1.65	^***^	1.35	^***^				
			851	1.82	^***^	1.50	^***^				
			2072	1.32	^***^	1.06					
Procollagen-lysine,2-oxoglutarate 5-dioxygenase 2	PLOD2		633	3.25	^***^	2.78	^***^		5.55	3.13	Iron
Prohibitin	PHB		1668	0.85	^**^	0.95			0.52	0.48	
Prolyl 4-hydroxylase subunit alpha-1	P4HA1		1049	2.79	^***^	2.63	^***^		3.21	2.08	Iron
RNA-binding protein 4B	RBM4B		1438	1.33	^***^	1.19	^**^		0.95	0.77	
Stress-induced-phosphoprotein 1	STIP1		1011	0.79	^**^	0.97			0.67	0.66	
Triosephosphate isomerase	TPI1		1786	1.47	^***^	1.30	^**^		1.69	1.27	Glycolysis

In proteomic changes, the mean ratios of identified spots between control and compound-treated cells are listed. Non-repeated-measures ANOVA and Dunnett’s test for post hoc analysis were performed. In transcriptomic changes, the ratio between control and compound–treated cells are listed. If there are more than one probes corresponding to the protein, the mean ratio was calculated. *P < 0.05; **P < 0.01; ***P < 0.001.
