# Supplementary material for: Proteomic profiling reveals that collismycin A is an iron chelator
Source: Sci Rep. 2016 Dec 6;6:38385. doi: 10.1038/srep38385 (PMC5138588; doi:10.1038/srep38385)
Supplement: Supplementary Information [file srep38385-s1.pdf]

## Supplementary Information

# Proteomic profiling reveals that collismycin A is an iron chelator

Makoto Kawatani[1], Makoto Muroi[1], Akira Wada[2], Gyo Inoue[3], Yushi Futamura[1], Harumi Aono[1], Kenshirou Shimizu[1,4], Takeshi Shimizu[1],Yasuhiro Igarashi[5], Naoko Takahashi-Ando[3], and Hiroyuki Osada[1*]

[1]Chemical Biology Research Group, RIKEN CSRS, 2-1 Hirosawa, Wako, Saitama 351-0198, Japan
[2]Nonnatural Amino Acid Technology Team, RIKEN CLST, 1-7-22 Suehiro-cho, Tsurumi-ku, Yokohama, Kanagawa 230-0045, Japan
[3]Department of Applied Chemistry, Faculty of Science and Engineering, Toyo University, 2100 Kujirai, Kawagoe, Saitama 350-0815, Japan
[4]Department of Materials Science and Engineering, Graduate School of Engineering, Tokyo Denki University, 5 Senju Asahi-cho, Adachi-ku, Tokyo 120-8551, Japan
[5]Biotechnology Research Center and Department of Biotechnology, Toyama Prefectural University, 5180 Kurokawa, Imizu, Toyama 939-0398, Japan

*To whom correspondence should be addressed. Tel: +81-48-4679541; Fax: +81-48-4679542; E-mail: hisyo@riken.jp

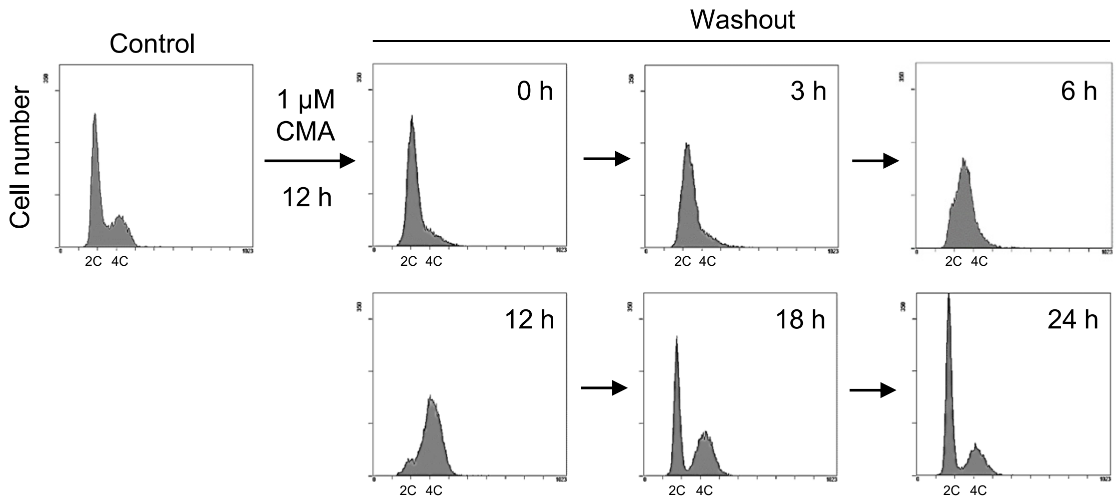


**Supplementary Fig. S1. Reversible effect of CMA on cell cycle progression.**
HeLa cells were treated with 1 μM CMA for 12 h, washed with PBS once, and then replaced into the medium without CMA. After replacement in the medium, cells were further cultured, and analyzed by flow cytometry after propidium iodide staining.

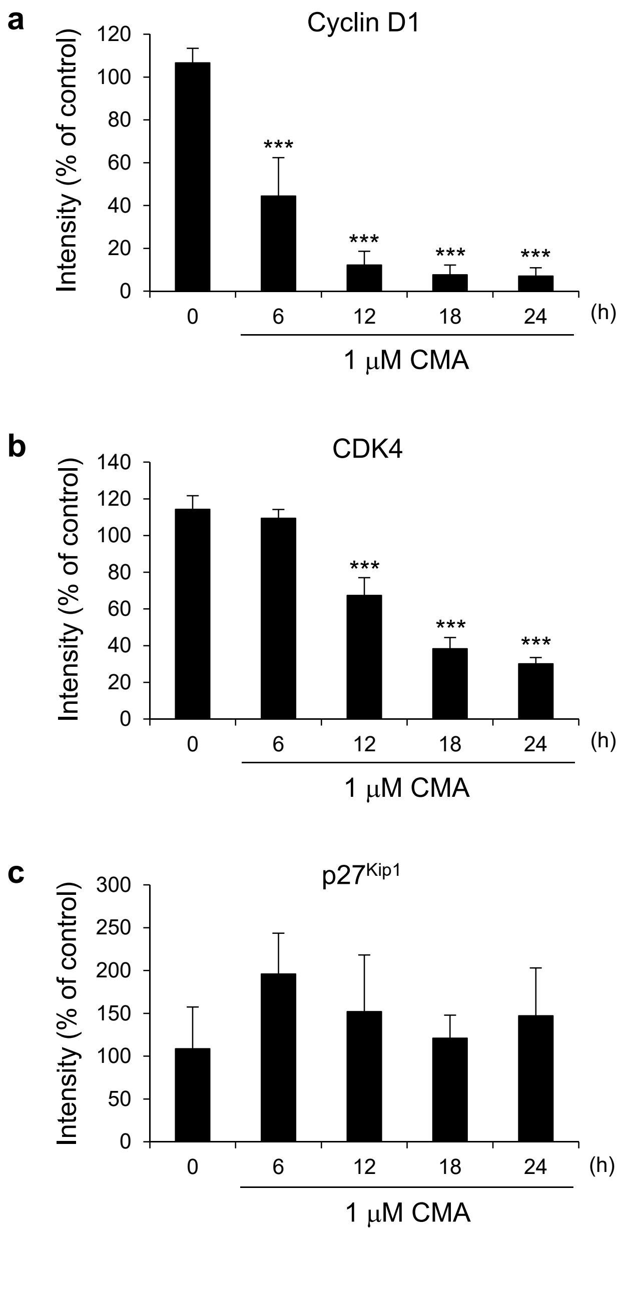


**Supplementary Fig. S2. CMA decreases the expression of cyclin D1.**
(**a**-**c**) HeLa cells were treated with 1 μM CMA for the indicated times. Cell lysates were immunoblotted with anti-cyclin D1, anti-CDK4, anti-p27$^{Kip1}$, and anti-α-tubulin (Fig. 1c). The band intensities were quantified using Fusion Solo S System (Vilber-Lourmat) and adjusted based on the corresponding loading control (α-tubulin). Data are shown as the mean ± SD (n = 3). Statistical analysis was performed by using ANOVA followed by Dunnett's test. ***, $P < 0.001$.

a

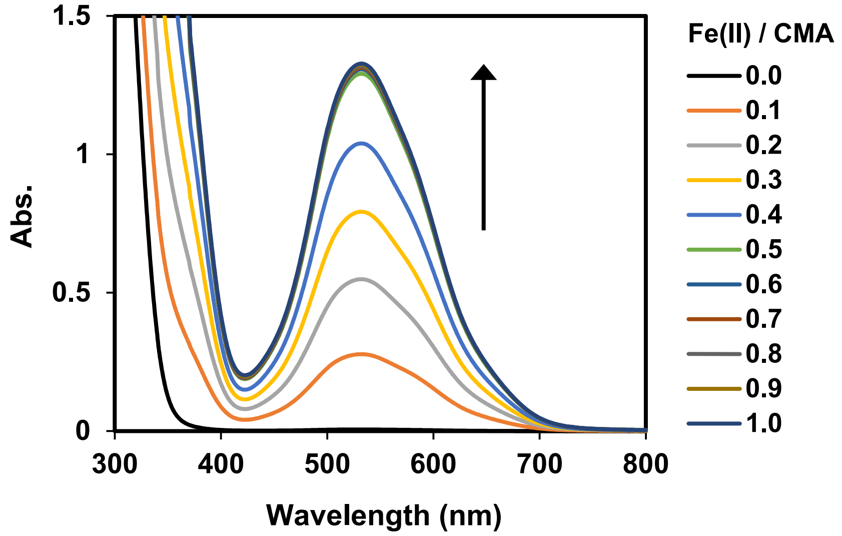


b

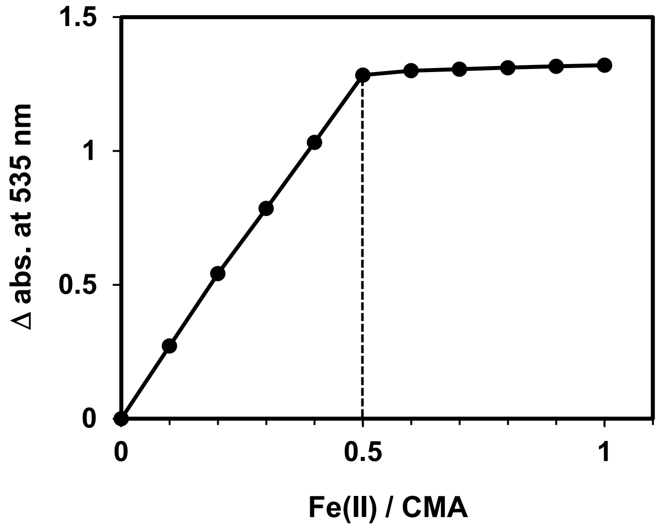


**Supplementary Fig. S3. Absorption spectroscopic analysis on the binding of CMA to Fe(II) ion.**

(**a**) Absorption spectra in the reactions of CMA (0.2 mM) with various concentrations of $FeSO_4$ in MOPS buffer (20 mM MOPS, 100 mM NaCl, pH 7.4, 10% MeCN). (**b**) Plotting of the absorption change at 535 nm against the molar ratio of Fe(II) / CMA for determining the binding ratio of CMA and Fe(II) ion. The binding of CMA to Fe(II) ion induced a new absorption band at 535 nm, and the intensity increased linearly upon incremental concentration of Fe(II) ion until the stoichiometric ratio of Fe(II) / CMA was reached to be 0.5. These spectroscopic data demonstrate the rapid and stable formation of 2:1 CMA-Fe(II) complex in solution, which has been identical to that determined by HPLC analysis (Fig. 3a).

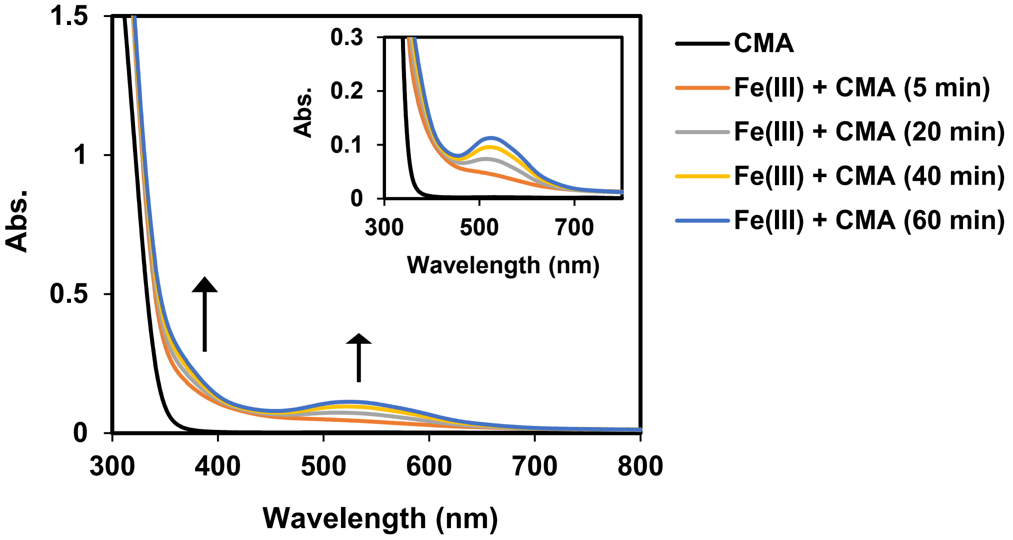


**Supplementary Fig. S4. Absorption spectroscopic analysis on the binding of CMA to Fe(III) ion.**

Absorption spectra in the reaction of CMA (0.2 mM) with $Fe_2(SO_4)_3$ (0.05 mM) in MOPS buffer (20 mM MOPS, 100 mM NaCl, pH 7.4, 10% MeCN) was measured by using V630 spectrometer (Jasco). A broad absorption in the range form 360 nm to 420 nm increased immediately after mixing CMA and Fe(III) ion, and no intense absorption appeared in visible region. This spectral change and pattern indicate a formation of Fe(III)-$(CMA)_2$ complex in solution, which has been supported by the similarity between Fig. 3a (left) and Fig. 3a (right) in HPLC analysis. Subsequently, a new absorption band around 530 nm increased slowly with time, which might reveal a gradual conversion of Fe(III)-$(CMA)_2$ complex to an another complex. To clarify this spectral change, further studies will be need.

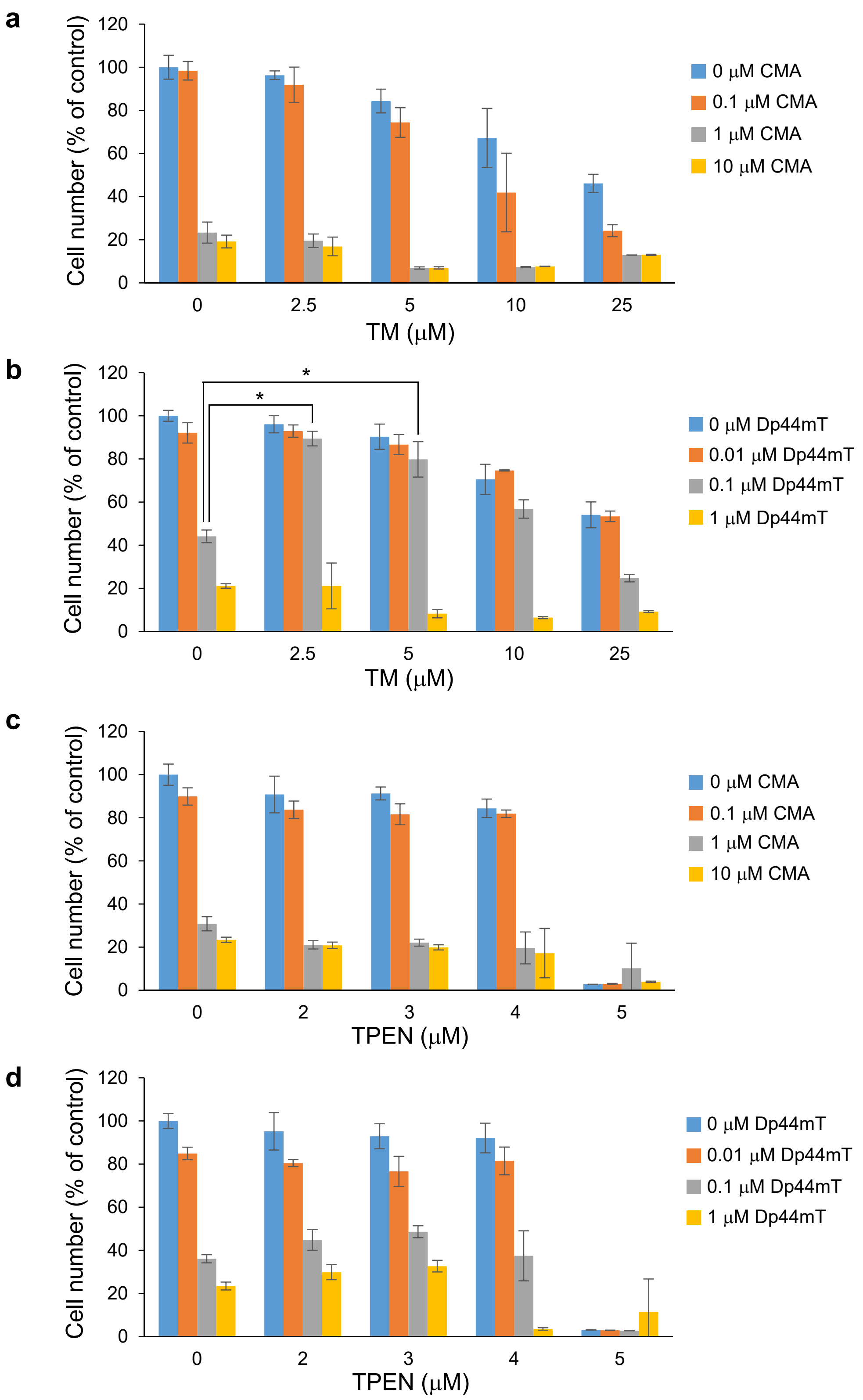


**Supplementary Fig. S5. Inhibition of cancer cell growth by CMA or Dp44mT in the presence of TM or TPEN.**

(**a-d**) HeLa cells were pretreated with TM or TPEN for 30 min, and then treated with CMA or Dp44mT for 72 h. Cell growth was analyzed by WST-8 assay. Data are shown as the mean ± SD (n =3). Statistical analysis was performed by using ANOVA followed by Tukey-Kramer test. *, *P* < 0.001.

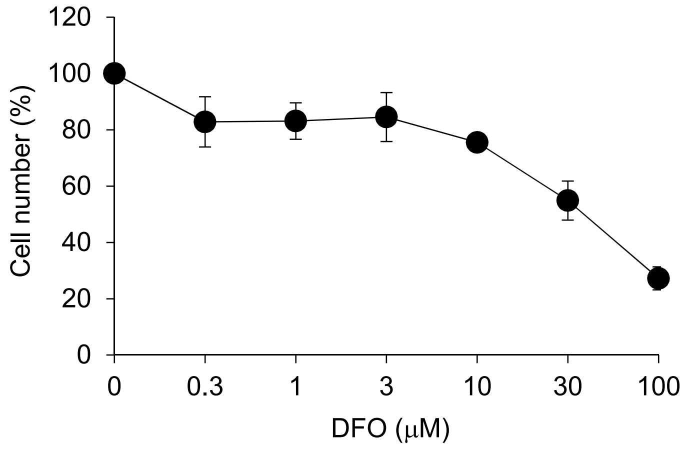


**Supplementary Fig. S6. DFO inhibits the growth of HeLa cells.**
HeLa cells were treated with DFO for 72 h, and cell growth was analyzed by WST-8 assay. Data are shown as the mean ± SD (n = 3).

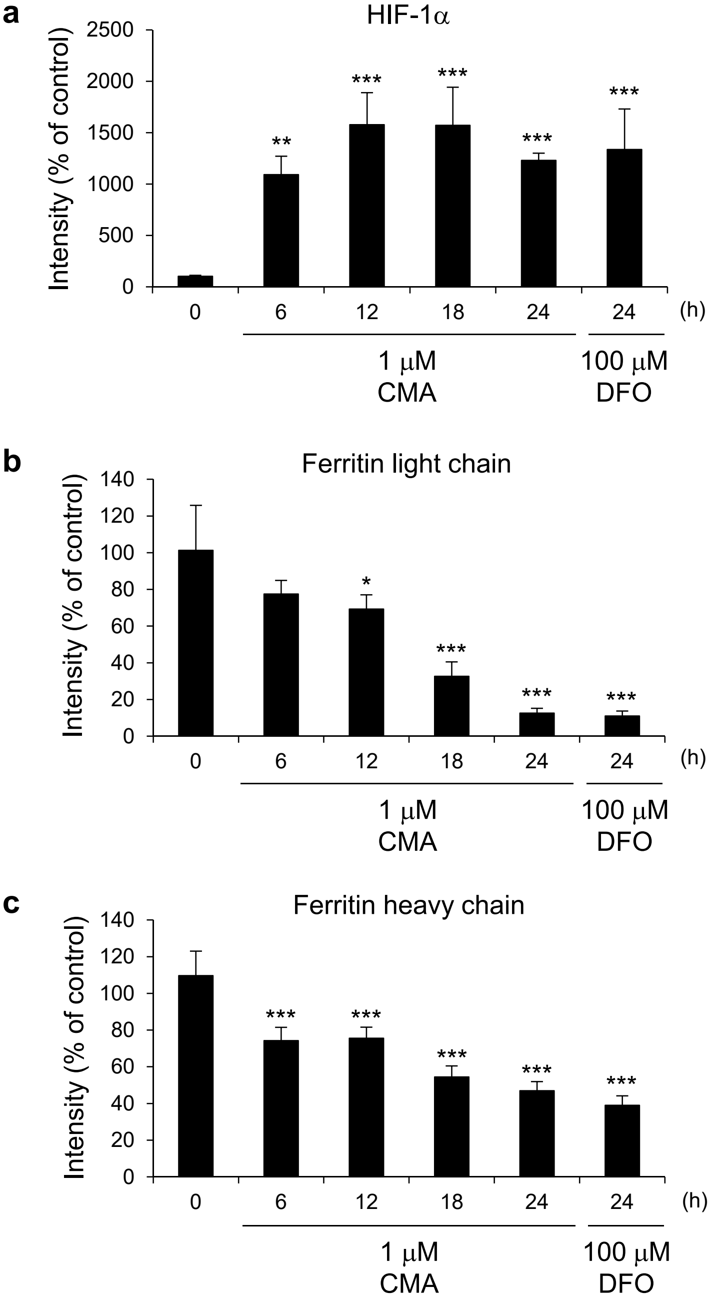


**Supplementary Fig. S7. CMA markedly induces the expression of HIF-1α.**
(**a-c**) HeLa cells were treated with 1 μM CMA or 100 μM DFO for the indicated times. Cell lysates were immunoblotted with anti-HIF-1α, anti-ferritin light chain, anti-ferritin heavy chain, and anti-α-tubulin (Fig. 4c). The band intensities were quantified using Fusion Solo S System (Vilber-Lourmat) and adjusted based on the corresponding loading control (α-tubulin). Data are shown as the mean ± SD (n = 3). Statistical analysis was performed by using ANOVA followed by Dunnett's test. *, $P < 0.05$; **, $P < 0.01$; ***, $P < 0.001$.

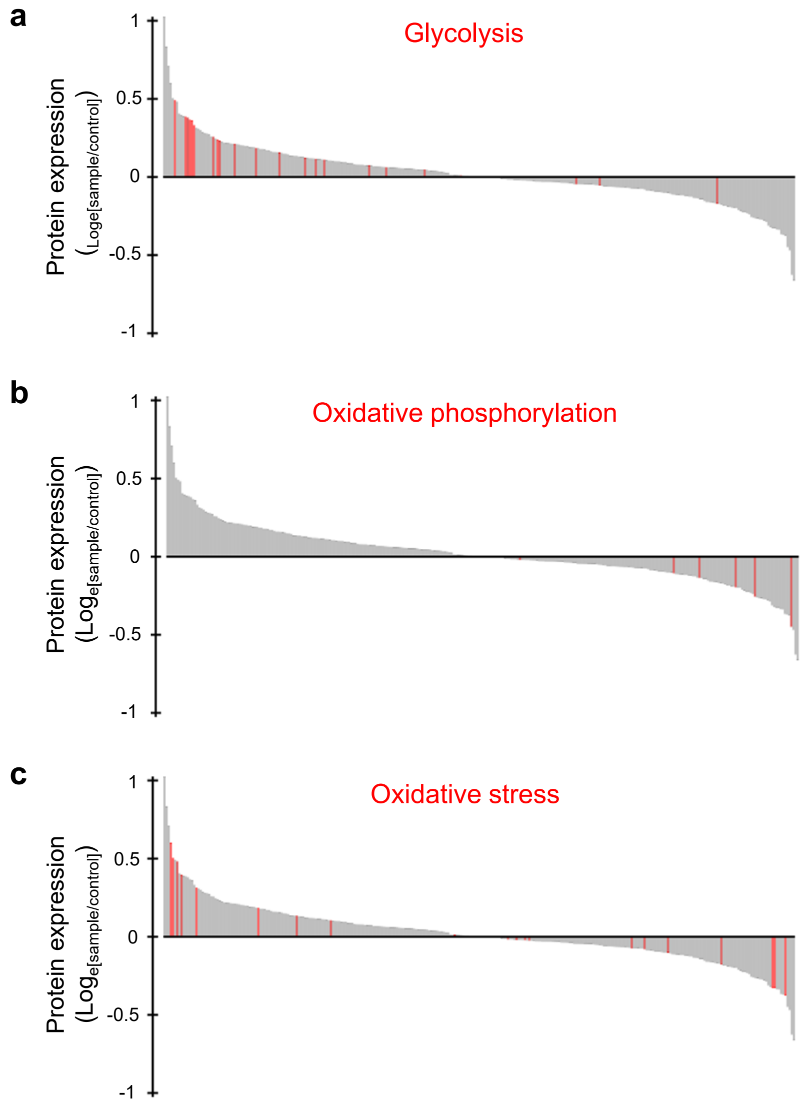


**Supplementary Fig. S8. Keyword based analysis of CMA-induced proteomic changes.**
(**a-c**) The log ratio values of spot expression between CMA-treated cells and control cells were plotted as a waterfall plot. The 296 bars were ordered in decreasing order of the log ratio of each spots. Two-hundred and seventy-four spots among the 296 spots were identified by peptide mass finger printing or LC-MS/MS. The bars corresponding to the protein spot which includes a keyword in the CC, DR, and KW lines of Uniprot database (http://www.uniprot.org) are indicated in red. The keywords are glycolysis (**a**), oxidative phosphorylation (**b**), and oxidative stress (**c**), respectively.

**Figure 1c**

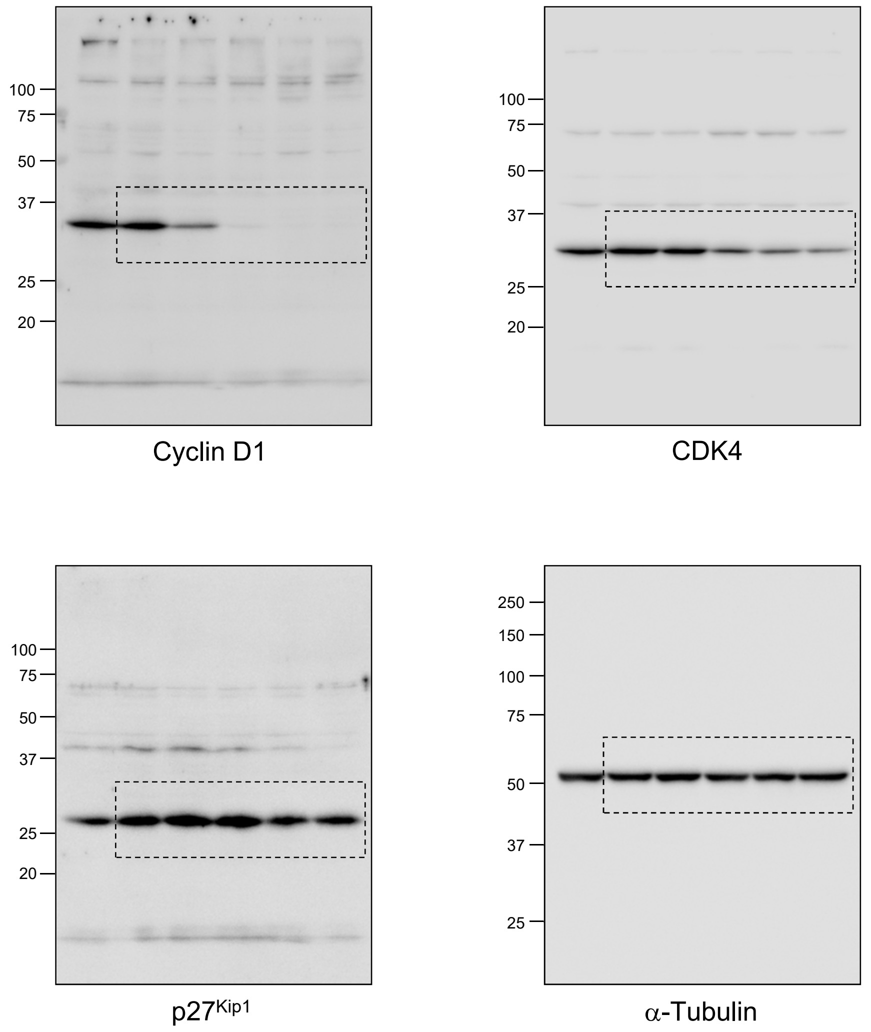


**Supplementary Fig. S9. Uncropped images of western blots accompanied by molecular size markers (kDa).**

**Figure 4c**

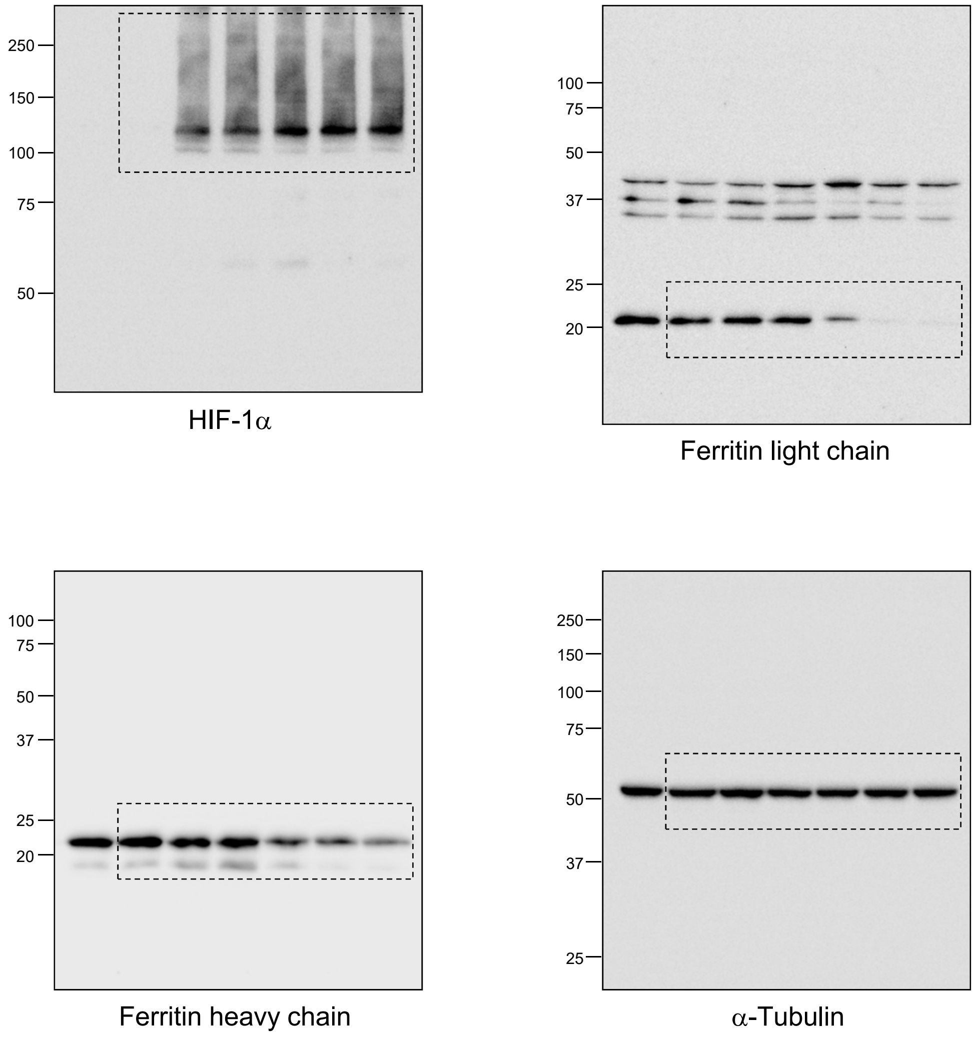


**Supplementary Fig. S9. (*continued*)**
